# A novel CARD11 germline mutation in a Chinese patient of B cell expansion with NF-κB and T cell anergy (BENTA) and literature review

**DOI:** 10.3389/fimmu.2022.943027

**Published:** 2022-09-20

**Authors:** Peiwei Zhao, Yanqiu Hu, Dongming Sun, Qingjie Meng, Lei Zhang, Xiankai Zhang, Li Tan, Yong Zhang, Yan Ding, Xuelian He

**Affiliations:** ^1^ Precision Medical Center, Wuhan Children’s Hospital (Wuhan Maternal and Child Healthcare Hospital), Tongji Medical College, Huazhong University of Science & Technology, Wuhan, China; ^2^ Department of Cardiology, Wuhan Children’s Hospital (Wuhan Maternal and Child Healthcare Hospital), Tongji Medical College, Huazhong University of Science & Technology, Wuhan, China; ^3^ Department of Clinical Laboratory, Wuhan Children’s Hospital (Wuhan Maternal and Child Healthcare Hospital), Tongji Medical College, Huazhong University of Science & Technology, Wuhan, China; ^4^ Rheumatology and Immunology Department, Wuhan Children’s Hospital (Wuhan Maternal and Child Healthcare Hospital), Tongji Medical College, Huazhong University of Science & Technology, Wuhan, China

**Keywords:** BENTA, *CARD11*, gain-of-function, lymphocytosis, NF-κB

## Abstract

Germline gain-of-function (GOF) mutations in the *CARD11* gene lead to a rare primary immunodeficiency disease known as B cell expansion with NF-κB and T cell anergy (BENTA). Affected patients present with a polyclonal expansion of B cells, lymphadenopathy, and splenomegaly. Herein, we report a novel germline in-frame three base-pair deletion (c.1030_1032del, p.K344del) in the *CARD11* gene in a patient with atypical BENTA, presenting with a recurrent fever and B cell lymphocytosis. This mutation was inherited from his mother, who is clinically asymptomatic and had a recurrent respiratory tract infection in her childhood. *In vitro* functional analysis demonstrated that this variant decreased the expression level of the CARD11 protein and activated the NF-κB signal pathway, leading to a higher expression of several NF-κB target gene transcripts in HCT116 cells transfected with mutant CARD11 (K344del-CARD11) as revealed by RNA sequencing analysis. To our knowledge, only 23 BENTA patients have been identified and carried seven distinct GOF mutations in *CARD11*. The clinical manifestations of patients are highly heterogeneous and there was no significant correlation between genotype and phenotype. In summary, we identified a novel in-frame three base-pair deletion that may be responsible for the pathogenesis of atypical BENTA in a Chinese family. Our study expands the mutational spectrum of the *CARD11* gene and may be helpful in the understanding of diseases caused by *CARD11* mutations and the clinical management of BENTA.

## Introduction

B cell expansion with NF-κB and T cell anergy (BENTA) (OMIM 616452) was first reported in 2012 and is a rare primary immunodeficiency disease. Patients present with persistent B cell lymphocytosis in early childhood, usually accompanied by lymphadenopathy and splenomegaly (1, 2). BENTA is caused by heterozygous germline gain of function (GOF) mutations in the *CARD11* gene, which encodes a caspase recruitment domain-containing protein (CARD11, also known as CARMA1) (3).

The CARD11 is a lymphocyte-specific scaffolding protein and acts as a critical signal transducer from the cell surface antigen receptor (AgR) in B or T cells to the cytoplasmic IκB kinase (IKK). This in turn activates the canonical NF-κB pathway (4). An overactive NF-κB pathway is associated with B cell malignancy (5). Somatic mutations in *CARD11* have been reported in different cancers, especially in diffuse larger B cell lymphoma (DLBCL) (6). Germline *CARD11* mutations have been associated with several primary immune disorders, including immunodeficiency 11A (OMIM 615206) (7), immunodeficiency with atopic dermatitis (OMIM 617638) (8), and BENTA caused by heterozygous GOF mutations (3). These immune disorders were caused by bi-allelic loss-of-function (LOF) mutations, heterozygous dominant negative, and GOF mutations, respectively. To date, 23 patients with BENTA have been identified to carry seven distinct GOF mutations, including C49Y, G123S, G123D, G126D, E134G, H234Ldel235-8, and K215del (3, 9–19). These mutations are located in the CARD, LATCH, or CC domains of CARD11.

In this study, a germline heterozygous mutation (c.1030_1032del, p.K344del) has been identified in the *CARD11* gene in a Chinese boy with recurrent fever and B cell lymphocytosis. This mutation was inherited from his mother who had a recurrent respiratory tract infection in childhood and is clinically asymptomatic. Our functional study suggests that this mutation resulted in an overactive NF-κB *in vitro*. In addition, we review the literature and summarize the clinical phenotypes and genotypes of all BENTA patients.

## Material and methods

### Patient and samples

This work has been approved by the ethics committee of Wuhan Children’s Hospital, Tongji Medical College, and Huazhong University of Science & Technology, and informed consent was obtained from the parents of the patient. The patient was recruited in this study because of recurrent fever. After 3ml of peripheral blood was collected from both this patient and his parents, genomic DNA and RNA were extracted by using the MicroElute Genomic DNA Kit (OMEGA Bio-tek) and Trizol reagent (Invitrogen), respectively.

### Whole exome sequencing

Trio WES was conducted with the help of the third-party medical testing laboratory (Chigene (Beijing) Translational Medical Research Center. China), and subsequent bioinformatic analysis was done as described previously in our laboratory (20).

The candidate gene variants were validated by Sanger sequencing in this patient and his parents. The primers sequence are as follows: CARD11-F: CAA CAG TCA GAT AGT CGG TTCC; CARD11-R: GAC AAA ACA CTC TGA AGG AGCC. The conservation analysis of the protein amino acid was conducted using MEGA software.

### *CARD11* gene plasmid construction and cell transfection

Site-directed mutagenesis was performed to generate K344del-CARD11 using specific primers for linear amplification, followed by DpnI digestion of methylated DNA. Positive colonies were subjected to sequencing analysis to verify the successful deletion of the guide sequence. The wildtype CARD11 plasmid (WT-CARD11*)* and positive control (C49Y-CARD11) were included as described previously (16).

After being cultured in DMEM supplemented with 10% fetal bovine serum (Gibco, Thermo Fisher Scientific) for 24 hours, HCT116 cells were transfected with 2 μg plasmids (pcDNA3.1, WT, C49Y, WT+K344del, and K344del using Lipofectamine 3000 (Invitrogen) according to the manufacturer’s instructions.).

### Western blot

The detailed experimental protocol is similar to our previously published article (16). Cells were lysed in NP-40 lysis buffer on ice, and then centrifugated at 12000rpm for 10 minutes at 4°C. Cell nucleoprotein was extracted using NE-PER™ Nuclear and Cytoplasmic Extraction Reagents (Thermo Fisher Scientific). The extracted protein was separated by SDS-PAGE and WB was used to detect the expression level of related proteins with the following antibodies: anti-CARD11(Proteintech, 21741-1-AP), anti-NF-κB p65(Cell Signaling Technology, #8242), lamin B (Santa cruz, sc-377000), and GAPDH(Proteintech, 60004-1-Ig).

### Immunofluorescence

Cells cultured in a confocal dish were fixed for 15 minutes with 3% paraformaldehyde and then permeabilized for 15 minutes in 0.1% Triton X-100/PBS. After blocking in a 3% BSA buffer, cells were incubated with the CARD11 antibody (Proteintech, 21741-1-AP) for 90 minutes. Cells were washed in PBS and then incubated with a secondary antibody conjugated to Alexa Fluor 488 (Invitrogen) at normal temperature. After washing three times, DAPI was added to the cell culture dish. Fluorescent images were obtained on a confocal microscope (Leica Stellaris 5).

### IgH rearrangement analysis

DNA was extracted from peripheral blood using MicroElute Genomic DNA Kit (OMEGA Bio-tek). PCR was performed using fluorescent-labeled primers to the V_H_ framework region (FR II) and joining region(J_H_) of the *IgH* gene. The amplification products were separated by capillary electrophoresis and analyzed on Genetic Analyzer (3500DX, ABI) using GeneMapper software version 5.0 (Applied Biosystems).

### Luciferase reporter assays

HCT116 cells were plated in 12-well culture plates (3 × 10^5^ cells/well) for 12h. The cells were then co-transfected with either 500 ng of pcDNA3.1+, CARD11-WT, and -C49Y- -K344del, 300 ng of pNFκB-luc (Bayotime, China) containing NF-κB binding motifs, or 100 ng of pRL-TK vector as a control (Promega, Madison, WI). After 36h, cells are lysed in lysate buffer and then analyzed for luciferase activity using the Dual-Luciferase reporter Assay System (Promega, Madison, WI). Three independent experiments were performed to assess luciferase activity.

### RNA-seq analysis

The experimental protocol was carried out as described previously (16). RNA was isolated from HCT116 cells transfected with CARD11-K344del or CARD11-WT plasmids, respectively. After constructing cDNA libraries, sequencing analysis was carried out on an Illumina sequencer instrument. Gene expression was calculated as transcripts per million (TPM) mapped reads by using the TopHat alignment program with redundant reads removed, and the expression values were normalized by using ${\text{Log}}_{2}^{\text{TPM}}$ . The raw data was submitted to the SRA database (SUB11737083).

### Quantitative PCR analysis

After the cDNA was generated from the total RNA isolated from HCT116 cells transfected with wild-type and mutant *CARD11* gene, respectively, a quantitative PCR experiment was performed on ABI 7500 instrument (Applied Biosystems, USA) using SYBR green preMix Taq Kit (TAKARA, Dalian). Single amplicons of predicted size were confirmed by agarose gel electrophoresis analysis. Raw data was calculated as an average of three independent experiments. *ACTIN* was included as an internal control.

### Statistics methods

Graphpad Prism 5 (Graphpad software, USA) was used for statistical analyses. The Student’s t-test was used to compare two groups, a one-way analysis of variance (ANOVA) was used to compare one variable among three or more groups, and statistical significance is indicated by a P-value (*P<0.05, **P<0.01, ***P<0.001). All experiments were performed in duplicates and repeated three times, and data are described as the mean ± SD.

## Results

### Case presentation

**Figure 1:**
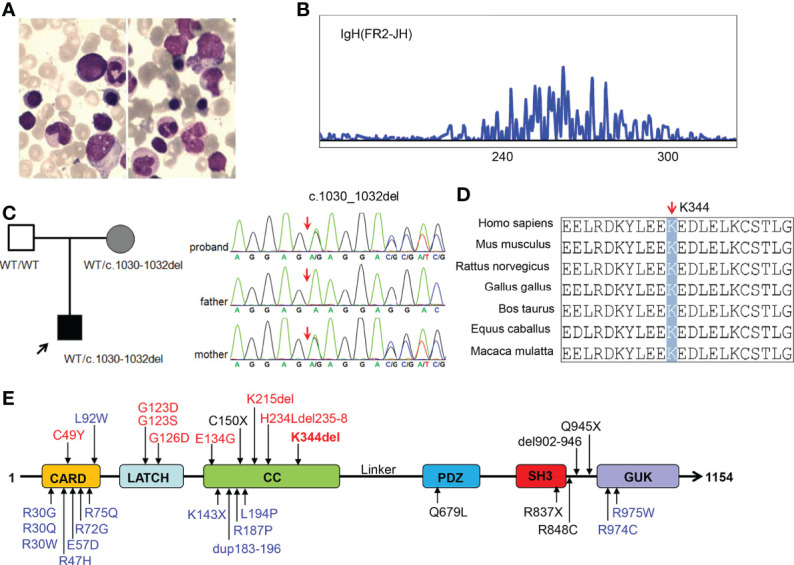
Characterization of our patient with *CARD11* gene mutation and conserved features of the CARD11 protein. **(A)** Bone marrow smear from the patient showing microcytic hypochromic anemia; **(B)** Result of IgH rearrangement of our patient; **(C)** Pedigrees of this family and sanger sequencing of *CARD11* mutation in this family; **(D)** Aligned amino acid sequence at this mutation among different species. The position at residue 344 is noted by a gray bar and highly conserved throughout all indicated species; **(E)** Scheme of the distribution of the CARD11 gain-of-function mutations, and the mutation noted in red was reported in this study. Gain of function mutations are highlighted in red, dominant negative mutations are in blue, and loss of function mutations are in black.

The patient was a 13-month-old male of Chinese descent who was referred for genetic evaluation because of unexplained recurrent fever. He is the only child born to healthy non-consanguineous parents without familial history of genetic diseases. There were no complications during pregnancy and no obvious abnormality was found at birth. When he was 7 months old, the patient presented with a recurrent fever (8 times within two months), with a maximum temperature of 39.2°C, and a persistent upper respiratory tract infection. He had a small rash on his face after the fever. The rest of his physical examination was unremarkable and he had no palpable lymph nodes. Clinical laboratory examination showed the proportion of lymphocytes (84.8%, normal range 40-70%) increased significantly with normal morphological characteristics and normal total white blood cell counts (9.67*10^9^/L). The CD19+ B cell population was expanded at presentation (3,432 cells/μL, normal range 240-1,317cells/μL), and the proportion of NK cells was decreased (3.03%). The patient did not receive any special treatment, other than immunization and other symptomatic treatments. Over the next six months, the patient still had periodic fever episodes occurring every 8 days and lasting for 2-3 days. When the patient was 13 months old, he was admitted to our hospital again because of recurrent fever. The summary of routine blood tests and immunological investigations is provided in **Table 1**. Initial blood analyses showed the proportion of lymphocyte cells and CD19+ B-cells were increased, and the hemoglobin level was decreased (90g/L, normal range 110-149g/L). A bone marrow biopsy showed hyperplastic anemia (**Figure 1A**). An IgH rearrangement experiment indicated that the patient’s B cells were polyclonal (**Figure 1B**). An abdominal CT scan showed multiple mesenteric lymphadenopathies and an ultrasound examination revealed a normal spleen size and an accessory spleen at the hilum.

**Table 1:** Clinical characteristics of the patient.

Clinical manifestation	Detection result	Reference value
Age/sex	13 month/male	
Age of onset	4 month	
Clinical manifestation	Recurrent fever; Hypogammaglobulinemia	
Glutamic-pyruvic transaminase (U/L)	14	9-60
Aspartate aminotransferase (U/L)	38	10-50
Ferritin (ng/ml)	11.34	22-322
WBC(10^9/L)	6.21	5.5-12
RBC(10^12/L)	4.74	3.7-5.3
PLT(10^9/L)	506	100-378
HGB(g/L)	90	110-149
Lymphocytes (%)	72.6	40-70
CD3+ T lymphocytes (n/ul)	5196	805-4459
CD3+%	46.16	38.56-70.06
CD4+ T lymphocytes (n/ul)	2526	345-2350
CD4+%	22.89	14.21-36.99
CD8+ T lymphocytes (n/ul)	2145	314-2080
CD8+%	19.43	13.24-38.53
CD 19+ B lymphocytes (n/ul)	5283	240-1317
CD 19+ %	46.08	10.86-28.03
CD 16 + 56+ NK cells (n/ul)	511	210-1514
CD 16 + 56+ %	4.45	7.92-33.99
s.IgG (g/L)	4.70	3.48-7.01
s.IgA (g/L)	< 0.26	0.28-1.08
s.IgM (g/L)	0.26	0.42-1.73
Triglycerides (mmol/L)	1.82	0.32-1.46
Bone marrow cell morphology	microcytic hypochromic anemia	

### Identification of germline CARD11 mutation

In order to determine the cause of the patient’s recurrent fever and rule out the genetic disorder, a trio WES was conducted. Bioinformatic analysis was performed to identify candidate variants according to filtering strategy and the pathogenicity of identified variants was assessed as described previously (20). After analysis, a heterozygous in-frame three base-pair deletion mutation (c.1030_1032del, p.K344del) was identified in the *CARD11* gene (NM_032415), and a Sanger sequencing analysis showed this mutation was inherited from the patient’s mother (**Figure 1C**). The mutated site is conserved among different species (**Figure 1D**). Interestingly, similar to most other BENTA-associated variants, the K344del variant was located in CC domain (**Figure 1E**). The germline variant has not been listed in the Clinvar, ExAC, gnoMAD, or dbSNP databases, while the somatic variant was included in the Catalogue of Somatic Mutations in Cancer (COSMIC) in a patient with diffuse large B cell lymphomas (DLBCL). We submitted this variant to the Clinvar database (https://submit.ncbi.nlm.nih.gov/subs/clinvar_wizard/SUB11735856/overview).

Given that the K344del variant was inherited from the mother, we also made a detailed inquiry into whether the mother had similar symptoms. The mother, who is clinically asymptomatic, told us that she had a recurrent respiratory tract infection in childhood. The clinical laboratory examinations, including the total white blood cell counts, the proportion of lymphocytes, CD19+ B cell population, and the proportion of NK cells were in normal ranges.

### K344del spontaneously aggregates and constitutively activates NF-κB

**Figure 2:**
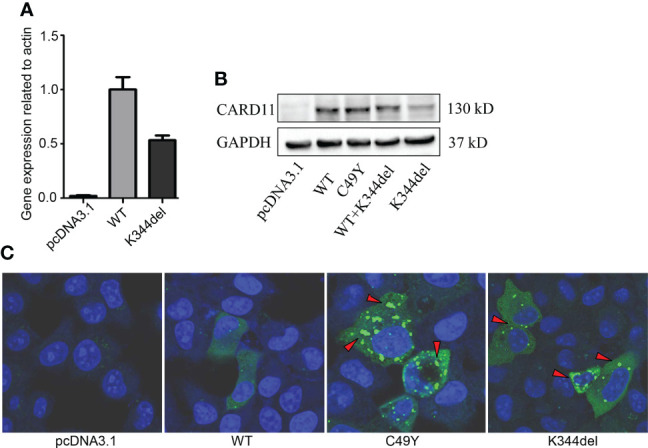
Expressions and Distribution of CARD11-WT and K344del in HCT116 cells. Expressions of RNA and protein of mutant CARD11 and its controls (**A, B**, respectively); **(C)** Distribution of CARD11-WT, -C49Y, and –K344del in HCT116 cells investigated by immunofluorescence.

**Figure 3:**
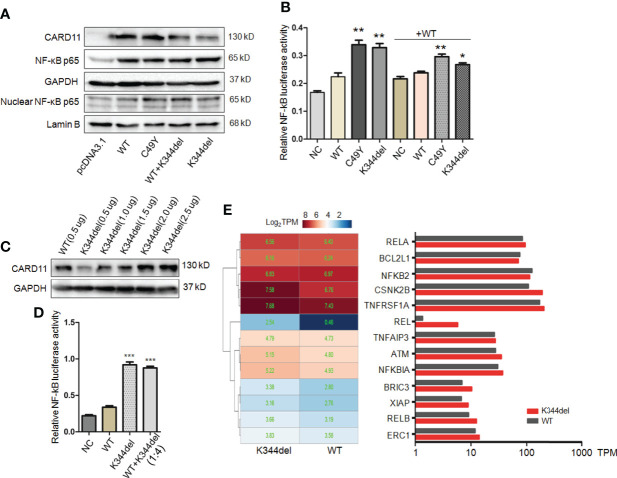
Functional analysis of K344del mutation in HCT116 cells. **(A)** The expression levels of CARD11, NF-κB p65 in HCT116 cells transfected with wildtype or mutant CARD11 in whole cell lysates and nuclear lysates, GAPDH and Lamin B serve as a loading control; **(B)** The activity of NF-κB-dependent luciferase of cell extracts from each sample was measured and recorded as a fold increase compared to control cells with WT-CARD11 plasmid. **(C)** The expression levels of CARD11 in different ratios of plasmid transfection. **(D)** The luciferase activity of cells transfected with mutants was increased in the case of consistent expression levels. The results from three independent experiments are described as the mean + standard deviation (*P < 0.05; **P < 0.01, ***P < 0.001). **(E)** Heat map of differentially expressed genes involved in NF-κB signal pathway between WT and K344del by RNA-seq analysis in HCT116 cells, the color scales of heatmap refer to Log_2_TPM.

To examine the effect of K344del, K344del-CARD11, positive control C49Y-CARD11, WT-CARD11, and empty plasmids were individually transfected into HCT116 cells. Our results showed that K344del significantly decreased the expressions of both gene and protein of CARD11 (**Figures 2A, B**). Similar to the positive control (C49Y), K344del resulted in CARD11 protein aggregation into large complexes, whereas WT-CARD11 dispersed throughout the cytoplasm (**Figure 2C**). In addition, p65 protein was significantly increased in mutant CARD11 compared to wild-type CARD11(**Figure 3A**). NF-κB activation was also examined by measuring pNF-kB-luc reporter gene expression in HCT116 cells transfected with pcDNA3.1(+), CARD11-WT, -K344del, or -C49Y plasmids. As shown in (**Figure 3B**), the luciferase activity in cells with mutated K344del-CARD11 was significantly higher than that in cells with CARD11-WT. Taken together, overexpression of K344del drives NF-κB activation. To mimic the proband’s state, K344del CARD11 was co-transfected at a 1:1 ratio with wild-type CARD11, and a similar change in the NF-κB signaling pathway was also detected in a heterozygous state by both western blot and luciferase reporter gene experiments (**Figures 3A, B**). Given that the cells only transfected with wild-type CARD11 had a higher level of CARD11 compared to those with only K344del-CARD11, a more appropriate ratio of WT-CARD11 to K344del-CARD11 used for transfection was determined to rule out the effect of wild-type CARD11. We found that the expression levels of wild-type and mutant CARD11 were comparable when the ratio of wild-type to mutant plasmid was 1 to 4 (**Figure 3C**). In the heterozygous state, similar to the proband, the activity of NF-κB luciferase was elevated significantly in the cells carrying the K344del plasmids *in vitro* (**Figure 3D**), suggesting that the mutation does not have a dominant negative effect. In addition, we also noted that several NF-κB target gene transcripts had higher expressions in HCT116 cells transfected with mutated CARD11 compared to that of wild-type CARD11 detected by RNA-seq (**Figure 3E**).

### Literature review of patients with BENTA

A literature review of BENTA cases was conducted by searching for all cases published from 2012 (BENTA was first reported in 2012) to 2021 with the keywords “*CARD11* gene” and “B cell expansion with NF-κB and T cell anergy “. The database included Pubmed, Medline, and Clinvar et al. We reviewed 12 articles which included 23 cases with BENTA, and the clinical features of these patients were summarized in **Table 2**.

**Table 2:** Literature review of the clinical features of BENTA patients.

Patients	Age/sex	lymphadenopathy	splenomegaly	lymphocytosis	Infections	IgG (g/L)	IgA (g/L)	IgM (g/L)	IgE (U/mL)	CD19+B	CD3+T	CD4+T	CD8+T	CD16+CD56	CARD11
P1[13]	8M/F	ND	√	√	EBV	6.9	0.06	0.43	312	46060	8751	5527	2303	921	G123S
P2[13]	2M/M	√	√	√	Sinopulmonary	32	3.09	3.89	1190	12027	4123	2405	1374	687	G123D
P3[13]	9M/F	√	√	√	Sinopulmonary	11.8	0.07	0.43	35.6	26150	5490	2906	1938	646	C49Y
P4[13]	3Y/F	√	√	√	Sinopulmonary,otitis media	12.7	0.08	1.45	4.45	1946	1342	940	268	34	C49Y
P5[3]	55/M	√	√	√	flu-like symptoms, pleuraleff usion.	hypogammaglobulinemia	low	low	–	–	–	–	–	–	E134G
P6[3]	13Y/F	√	ND	√	frequent upper respiratory tract, throat, and middle ear infections. She also had occasional sinus infections	hypogammaglobulinemia	–	low serum IgM	–	4703	2287	1202	697	268	E134G
P7[3]	11Y/F	√	√	√	recurrent tonsillitis and otitis media.	–	–	low serum IgM	–	4710	2464	1282	885	325	E134G
P8[3]	6Y/F	√	√	√	bronchitis, bilateral pneumonia with Streptococcus pneumoniae bacteremia	–	–	–	–	8105	6229	1384	4230	1061	G123S
P9[10]	22M/F	√	√	√	coryzal symptoms and intermittent diarrhea	9.46	0.58	0.73	92.6	7160	3630	2480	7600	460	K215del
P10[11]	80Y/F	ND	ND	ND	suffered from recurrent warts, shingles and sinusitis	–	–	–	–	–	–	–	–	–	H234LΔ235-8
P11[11]	57Y/F	–	–	√	frequent otitis externa and colds; persistent onychomycosis	11.35	2.55	0.37	16	612	2182	1074	970	306	H234LΔ235-8
P12[11]	32Y/M	–	–	√	multiple warts on hands, EBV	11.29	1.61	0.39	37.5	1066	2007	1328	535	197	H234LΔ235-8
P13[11]	6Y/M	N	N	√	pustular psoriasis, pneumonia	12.04	0.72	0.14	136	1602	1961	1291	507	133	H234LΔ235-8
P14[12]	59Y/M	√	√	√	suspected viral meningitis, pneumonia, childhood	–	–	–		3600	1500	1300	150	–	G123S
P15[14]	20Y/F	ND	√	√	fever, urticaria followed by septic infection	5.55	0.27	0.62		~5000	~580	~300	~220	–	G123S
P16[15]	12Y/M	√	√	√	otitis media, febrile episodes, episodes of sinusitis and bronchitis	–	–	–		48262	5522	2651	1877	1270	G123D
P17[16]	8M/M	√	√	√	a persistent upper respiratory tract infection, fever with splenomegaly	18.6	0.11	0.52		2882	1624	648	932	372	G126D
P18[19]	12Y/F	√	√	√	respiratory tract infections, cervical lymphadenopathy, and tonsillitis	5.01	1.69	0.37	1	higher than normal	–	–	–	–	G123S
P19[17]	43Y/F	ND	ND	√	EBV	–	–	–		1142	–	–	298	96	C49Y
P20[18]	16Y/F	ND	√	√	upper respiratory tract	7.56	0.27	0.75		2801	999	592	342	283	C49Y
P21[18]	18Y/M	√	√	√	recurrent URT infections, otitis media,	9.93	0.6	1.38		1900	1672	1102	418	228	C49Y
P22[18]	51Y/F	N	N	√	N	11.13	1.39	0.32		912	798	513	152	190	C49Y
P23[9]	13Y/F	√	√	√	adenopathy. recurrent otitis media	normal	normal	normal		higher than normal	–	–	–	–	C49Y
This work	13M/M	√	N	N	Recurrent fever; hypogammaglobulinemia	4.70	0.26	0.26		5283	5196	2526	2145	511	K344del

ND, no data.

Among the 24 BENTA patients (17 families), there were 9 male patients and 15 female patients, aged from 2 months to 80 years. The main clinical manifestations in the patients were lymphocytosis, splenomegaly, lymphadenopathy, recurrent infection, and recurrent fever.

The clinical presentation and course are highly variable, from severe lymphocytosis and dying at a very young age (Tab, P1, P3, P4), to repeated infection in childhood and clinically asymptomatic in adulthood (P22). A total of 7 germline GOF mutations (C49Y, G123S, G123D, G126D, E134G, K215del, and H234Ldel235-8) have been reported (8–13), and these mutations are located in the CARD, LATCH, or CC domains of CARD11 (**Figure 1E**). The K344del reported in this study is located in the CC domain. There was no significant correlation between genotype and phenotype. For instance, Buchbinder reported that 3 patients carrying C49Y mutation presented with milder clinical symptoms, while patients (P3 and P4) with this mutation died at a young age (13). All these variants, except E134G and H234Ldel235-8, were included in the COSMIC database and are identified as somatic mutations in patients with DLBCL, chronic lymphocytic leukemia, or lymphoma. This information indirectly indicates that BENTA patients with GOF mutation are at a high risk of developing lymphoma or leukemia, and some BENTA patients had developed lymphoma or B-ALL in adulthood (3, 11).

## Discussion

In this work, we have reported on a 13-month-old patient with B cell lymphocytosis, persistent recurrent fever, and upper respiratory tract infection but without lymphadenopathy or splenomegaly. The WES revealed a novel germline in-frame three base-pair deletion, c.1030_1032del(p.K344del) in the *CARD11* gene, which was associated with BENTA, severe combined immune deficiency, and severe atopic disease. According to the patient’s clinical manifestations, laboratory results of B cell lymphocytosis, and genetic findings, a diagnosis of atypical BENTA was considered.

The NF-κB family of transcription factors plays a crucial role in inflammation, immunity, and cell proliferation (5, 21). CARD11 is expressed mainly in lymphocytes and is a large scaffold protein bridging the AgR of B or T cells with several signaling pathways, including NF-κB (22). GOF mutants in the *CARD11* gene result in the spontaneous assembly of the CARD11-BCL10-MALT1 complex, which drives constitutive activation of NF-κB (23). Somatic K344del mutation was reported in one patient with diffuse large B-cell lymphoma (24), and to our knowledge, this variant has not been reported to be inherited (germline). To investigate the effects of K344del, we expressed the mutant and wild-type constructs along with a positive control (C49Y) in HCT116. Our findings showed that the K344del variant resulted in the aggregation of CARD11 and increased activation of NF-κB, consistent with previous studies on GOF mutations in the *CARD11* gene (16). Besides the GOF effect, CARD11 could lead to a dominant negative effect, involving immunodeficiency with atopic dermatitis. We noted that affected members in a four-generation family with a novel heterozygous germline mutation (c.701-713delinsT, p.H234Ldel235-8) in *CARD11* exhibited “mixed” clinical manifestations of BENTA (milder to moderate B cell lymphocytosis) with atopic dermatitis, which is consistent with both the GOF and dominant negative signaling effects (11). To investigate if the K344del CARD11 has a dominant negative effect, indicated by decreased activity in the heterozygous state compared with wild-type, plasmids with K344del CARD11 were co-transfected at a 1:1 ratio (or 4:1 ratio) with wild-type CARD11. We found that the NF-κB signaling pathway was also activated in the heterozygous state and K344del did not have a dominant negative effect.

By reviewing the literature, a total of 23 patients were identified, and their clinical manifestations were highly heterogeneous. Previous studies reported that patients with G123D, G123S, or G126D mutation had more severe symptoms, including B cell lymphocytosis, immunodeficiency, or death at a young age (13, 15, 16). Patients possessing the C49Y variant could present with recurrent fever and mild infection, and/or fatal hemophagocytic lymphohistiocytosis, and have a high risk for mortality at a young age (13, 19). It is clear that GOF mutation within CARD and LATCH domains is associated with severe phenotypes but more studies are needed for further confirmation. The heterogeneity of clinical phenotypes could be due to the potency of the GOF effect, additional genomic variants or modifications, and environmental factors. In addition, recurrent infection also affects CARD11 activity and disease severity. Lastly, the structural biology of the mutant CARD11 within the CARD11-BCL10-MALT1(CBM) signalosome complex may be another factor to mediate the CARD11 activity. This in turn, affects clinical symptoms and needs further study. For the patients with BENTA that survive to adulthood, as B cell lymphocytosis gets milder with aging (14), it is likely that patients are clinically asymptomatic, like the mother of our patient, and could be missed.

Polyclonal expansion of B cells is a hallmark of BENTA and polyclonal expansion of B cells increases the risk of B cell malignancy later in life. In addition, BENTA patients also have recurrent infections, including Epstein–Barr virus (EBV) infections. Impaired control of EBV infection has emerged as a recurring problem in BENTA disease (18, 25). A previous study demonstrated that the control of viral infection was inadequate, especially of chronic, low-grade EBV viremia, and the presence of EBV viremia may also increase the risk of B cell lymphomagenesis (26). Therefore, BENTA patients should be regularly monitored for B cell clonal expansion and infection, even EBV viral load.

There are some limitations in our study. Due to the difficulty in obtaining specimens from the patient and his mother, an experiment to examine the NF-κB activity and cellular distribution of CARD11 was not done. In addition, the clinical laboratory examination regarding the presence or absence of B cell expansion was missing. Finally, *in vitro* functional study on the mutant CARD11 was performed in HCT116 cells but not in lymphocyte cell lines, even though the expression level of CARD11 was low in HCT116 cells (**Figures 2A, B**), which can reduce background interference.

In conclusion, we reported a germline heterozygous GOF variant in the *CARD11* gene from a patient with B cell lymphocytosis and constitutive NF-κB activation. Our study provides functional evidence of the pathogenicity of this mutation. The literature review indicates that the clinical manifestations of BENTA patients are highly heterogeneous. Additional basic research focusing on the CARD11 signal and the balance of CARD11 activity will help determine the factors that influence BENTA pathogenesis.

## Data availability statement

The datasets presented in this study can be found in online repositories. The names of the repository/repositories and accession number(s) can be found below: SRA PRJNA855490, ClinVar SCV002564141.1.

## Ethics statement

The studies involving human participants were reviewed and approved by Institutional review board of Wuhan Children’s Hospital, Tongji Medical College, Huazhong University of Science & Technology. Written informed consent to participate in this study was provided by the participants’ legal guardian/next of kin.

## Author contributions

Study concepts: XH, YD. Study design: PZ, DS, QM, YD, XH. Literature reserch: QM, PZ, YH. Clinical information collection: DS, QM, YH. Data acquisition: QM, XZ, LZ, YZ, YH. Data analysis/interpretation: LZ, QM, XZ, LT. Manuscript preparation: XH, PZ. Manuscript editing: XH. Manuscript final version approval: YD. All authors contributed to the article and approved the submitted version.

## Funding

This work was supported by grants from the Wuhan Municipal Health Commission (NO.WX19C19) and the Youth Program of National Natural Science Foundation of China (NO.81700302).

## Acknowledgments

We thank the patient for participating in this study.

## Conflict of interest

The authors declare that the research was conducted in the absence of any commercial or financial relationships that could be construed as a potential conflict of interest.

## Publisher’s note

All claims expressed in this article are solely those of the authors and do not necessarily represent those of their affiliated organizations, or those of the publisher, the editors and the reviewers. Any product that may be evaluated in this article, or claim that may be made by its manufacturer, is not guaranteed or endorsed by the publisher.
